# QSRR Automator: A Tool for Automating Retention Time Prediction in Lipidomics and Metabolomics

**DOI:** 10.3390/metabo10060237

**Published:** 2020-06-09

**Authors:** Bradley C. Naylor, J. Leon Catrow, J. Alan Maschek, James E. Cox

**Affiliations:** 1 Metabolomics, Proteomics and Mass Spectrometry Cores, University of Utah, Salt Lake City, UT 84112, USA; brad.naylor@cores.utah.edu (B.C.N.); leon.catrow@utah.edu (J.L.C.); alan.maschek@pharm.utah.edu (J.A.M.); 2 Department of Biochemistry, University of Utah, Salt Lake City, UT 84112, USA; 3 Department of Nutrition and Integrative Physiology, University of Utah, Salt Lake City, UT 84112, USA

**Keywords:** metabolomics, lipidomics, retention time prediction, machine learning, automation

## Abstract

The use of retention time is often critical for the identification of compounds in metabolomic and lipidomic studies. Standards are frequently unavailable for the retention time measurement of many metabolites, thus the ability to predict retention time for these compounds is highly valuable. A number of studies have applied machine learning to predict retention times, but applying a published machine learning model to different lab conditions is difficult. This is due to variation between chromatographic equipment, methods, and columns used for analysis. Recreating a machine learning model is likewise difficult without a dedicated bioinformatician. Herein we present QSRR Automator, a software package to automate retention time prediction model creation and demonstrate its utility by testing data from multiple chromatography columns from previous publications and in-house work. Analysis of these data sets shows similar accuracy to published models, demonstrating the software’s utility in metabolomic and lipidomic studies.

## 1. Introduction

Mass spectrometry (MS) is commonly used for metabolite and lipid profiling. MS allows the measurement of mass to charge rations (*m/z*) of hundreds of compounds in a single analysis. While incredibly useful, determining the identity of a compound only by its *m*/*z* can be difficult. The same *m*/*z* can belong to isobaric and isomeric compounds in the same organism [1,2,3,4] or can be artifacts caused by the MS [5]. The traditional response to this problem is to fragment the compounds of interest using collision induced dissociation (CID) and examine the fragment *m*/*z* values (MS/MS fragmentation). While effective in a number of cases many metabolites have similar fragments. Metabolite MS/MS libraries and fragment prediction software are not yet sufficient to identify all compounds solely by fragmentation [1,2,6,7]. To ensure the proper identification of compounds, observed orthogonal measurements are needed.

A common orthogonal method of compound identification is chromatographic retention time [1,4,6,8,9]. Retention time is determined by chemical interactions with the chromatographic column and the eluents used. Using this property, possible compound identities can be narrowed down in a reproducible and chemically relevant way. However, the difference between liquid chromatography (LC) systems, columns, eluents, and gradients can cause large differences between the retention time of the same compound under different conditions. This is especially true when performed by different labs, even when using the same type of LC system and chromatography column [3]. There are two main approaches to correct for this variation. One is to compare the same compound in different conditions to create a model to predict how the compound will perform in any given condition. This is done very well by the PredRet software [3]. The main limitation of this approach is the requirement to have measured the same compound in many different conditions [3]. New compounds, rarely seen compounds, or unusual columns or conditions are difficult or impossible to predict.

The other approach is Quantitative Structure Retention Relationships (QSRR) [10]. In QSRR many chemical standards (at least 50–100, though more are better if possible) are analyzed on a particular LC-MS method. The chemical features of the standards are used to create a model to predict retention times of new compounds with similar features. This has the advantage of theoretically allowing prediction of any compound so long as its structure is known. Several papers have been published demonstrating the validity of using QSRR in metabolomics and lipidomics studies using various columns and conditions [1,2,6,7,8]. They demonstrate that QSRR techniques work for predicting retention time of various compounds. However, QSRR models are specific to the column, eluents, and LC method used, so the QSRR model must be recreated using standard lipids or metabolites for every set of LC conditions required. This is problematic for some laboratories, such as core laboratories, which have multiple methods, columns, and instruments with many compounds to identify. In addition, not all laboratories have a bioinformatics specialist who can be dedicated to making new QSRR models for each different experimental condition. An automated system to assist is needed in such situations.

**Figure 1:**
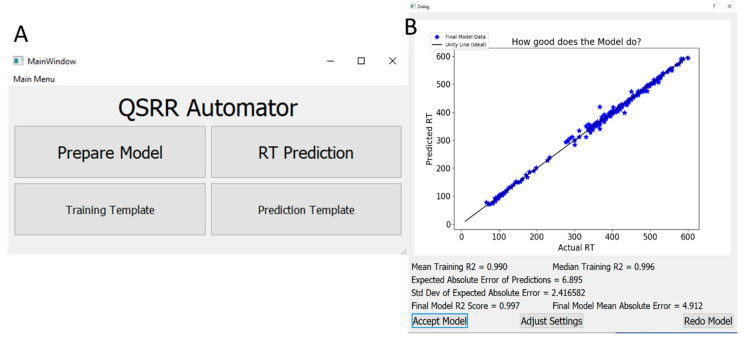
QSRR Automator (**A**). User interface for QSRR Automator. (**B**). Example model output of QSRR Automator using data from Aicheler et al. [1].

Here we present QSRR Automator, a user-friendly program that creates QSRR models for lipidomics and metabolomics which can be used by investigators with minimal training in bioinformatics (Figure 1). The software has been tested on LC-MS data collected from multiple experiments using multiple column types and eluents. While some of the benefits of expert model creation are lost, the ability to create multiple QSRR models quickly outweighs this disadvantage for a lab needing multiple models.

## 2. Results

### 2.1. Comparison to Previously Published Data

To ensure QSRR Automator was functioning appropriately, we collected data from multiple papers that had generated data from multiple types of compounds on different columns (details and references listed in Table 1). Most of the data sets were from metabolomics experiments that employed Hydrophilic Interaction Liquid Chromatography (HILIC) columns. Due to the more reproducible nature of Reverse Phase (RP) chromatography that is employed in many lipidomics studies, the creation of a QSRR model for this was a less difficult problem and required less testing [11,12]. All three models that QSRR Automator can use are represented in the published papers: Support Vector Machines for Regression (SVR) with a Radial Basis Function (rbf) kernel, Multiple Linear Regression (MLR) and Random Forest (RF).

#### 2.1.1. Direct Comparison of QSRR Automator to Published Data-Sets

To test how QSRR Automator performed on various data sets, the full data sets used in the creation of the published models were used to create QSRR Automator models. Because feature selection and model selection are affected by random data splits, five models were created for each dataset to ensure results were consistent. A comparison of the models presented in the published data and those created by QSRR Automator is presented in Table 1.

#### 2.1.2. Use of Published Test and Training Data-Sets

One danger of machine learning is overfitting the model to the training data, limiting the ability to apply the model to new data. The ideal way to determine if a model will predict future data well is to split data into a training and test set, with the training set being used to train the model and the test being “new” data on which performance can be evaluated. The cross-validations in QSRR Automator and the published data [1,2,6,7] are designed to prevent overfitting, but the possibility still exists. Since cross-validation functions report the average performance of each comparison rather than reporting all data, it makes individual training test comparisons between the published data-sets difficult to conduct. However, data-sets RP_Lipid, RP_Met, HILIC_MLR1 and HILIC_MLR2 provided a training and test set for their final models. This provides an excellent opportunity to compare QSRR Automator on a truly unseen testing sets and compare the results to published data.

**Figure 2:**
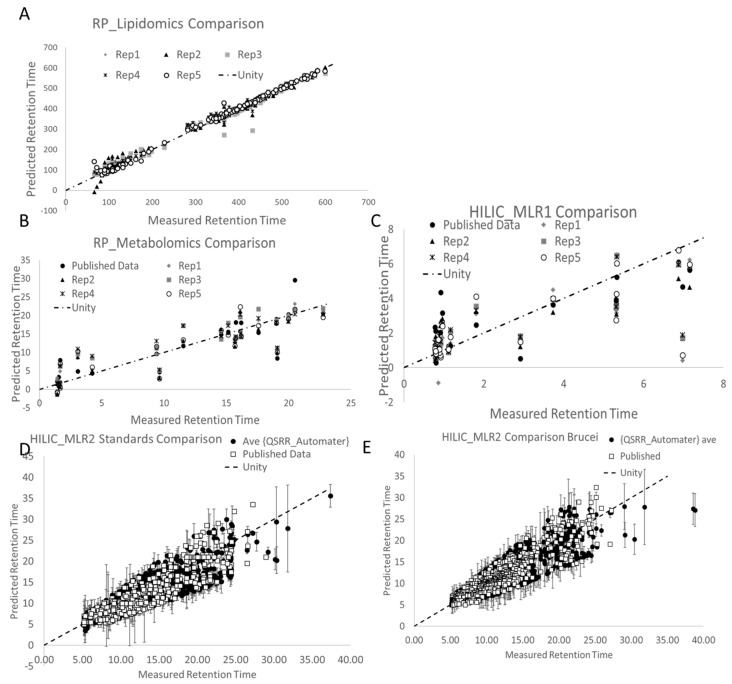
Comparison of QSRR Automator’s Predictions vs. Published. Predictions on published test sets. Unity lines show perfect predictions; Published Data are represented as are individual replicates (rep1-rep5). HILIC_MLR2 is represented with the arithmetic mean to aid readability. A perfect predictive model would put all points on the central unity line. This uses the RP_Lipidomics (**A**), RP_Metabolomics (**B**) HILIC_MLR1 (**C**), and HILIC_MLR2 datasets. HILIC_MLR2 used a mix of Standards (**D**) and an extract from Trypanosome Brucei (**E**). Details on these datasets are in Table 1.

Since random chance plays a role in feature and model selection in QSRR Automator and there is no human supervision of these processes, QSRR Automator was performed five times. The average of the QSRR Automator predictions and the measured standard values were compared to the difference between the published prediction and the true values. The results are shown in Figure 2.

Models in Figure 2 and their best fit equations in Table 2 show how QSRR Automator predictions compare to predictions made in various published studies. They were further analyzed in Tables S1 and S2. How well QSRR Automater does depends on the dataset being used, which may depend on the molecular features and compounds being used. Slopes are similar though r^2^ and intercept are where the worst errors are, both of which are likely influenced by more error in several points or a few large outliers. This suggests that while a dedicated bioinformatician can better shape the model to reduce the error, QSRR Automator produces similar results overall.

HILIC_MLR2 contains 2150 compounds with only 600 (standards) or 670 (brucei) compounds deemed potentially valid by the authors due to their filtering criteria [2]. The primary filter was dropping any prediction with an error greater than 35%. When predicting all 2150 compounds with QSRR Automator and applying the same filtering method, 650 compounds were deemed valid for both data sets (Table S1). As this is similar to the published values we can conclude QSRR Automator predicts equally well to the published method.

Finally, we compared the values predicted in the papers to those predicted by QSRR Automator using t-tests. We performed a paired t-test on the predictions of RP_Lipidomics, RP_Metabolomics, and HILIC_MLR1 on the assumption that the models should be predicting the same value as the true retention time, with all compounds being the same. We also compared each data set’s prediction to the measured values using t-tests again using paired t-tests on the assumption the same prediction should be the same and all compounds were identical. HILIC_MLR2 used normal t-tests since the data were not paired due to needing to recalculate which compounds to include. There is no evidence of statistical significance in any of the tests save for those involving HILIC_MLR2. *T*-tests are affected by the number of values (which is quite high in HILIC_MLR2) and there is a significant difference between published HILIC_MLR2 predictions and its measured data, so this likely does not indicate one model is superior to the other. Results are shown in Table S2.

### 2.2. Tests on In-House Data

#### 2.2.1. Lipidomics Data

**Figure 3:**
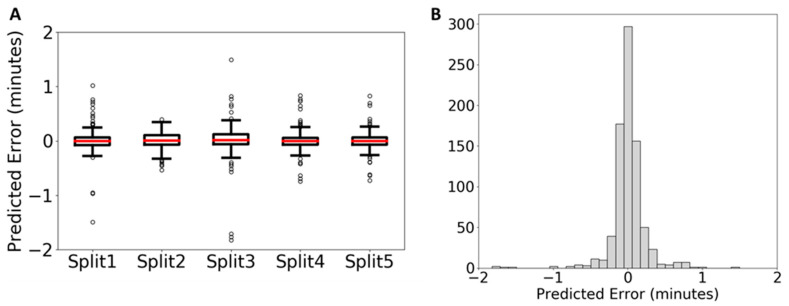
Analysis of lipidomics error. error of each prediction in absolute time is presented. This is presented with error divided by each test/training split (**A**) and the error of all compounds across all groupings (**B**) With all compounds (**B**) the *y*-axis represents the number of compounds at each error value.

Red blood cells were used for an in-house test of the capability of QSRR Automator for lipidomic retention time prediction. Lipids identified with their retention times are presented in Table S3. Samples were split into a training and test split and a model created. Five training/test splits were used each with three replicates to ensure that this analysis was truly representative of the performance of QSRR Automator. The prediction performance on the testing set is shown in Figure 3 with more data on the individual models present in Table S4. As can be seen in Figure 3, the error is clustered around zero with most of the error well within a minute. Outliers vary between the five test/training splits (Figure 3A) which indicates that the error is the result of random noise within the different training sets rather than a bias in the prediction software itself. As with the published analysis, more features were selected (selected features listed in Supplemental Table S5) though the majority of features are present only in some models with a smaller core of features used in all models.

#### 2.2.2. Metabolomics Data

**Figure 4:**
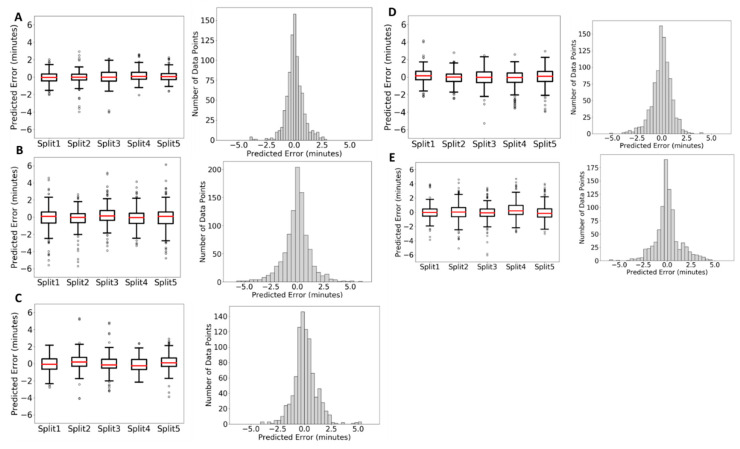
Analysis of error. Each plot has a box plot to show average error of points in separate test/training splits to show consistency and a histogram of all data points to show distribution of error throughout all samples. (**A**). BEH-Amide column, (**B**). CHILIC column, (**C**). HILIC-Z column, (**D**). iHILIC column, (**E**). PHILIC column.

To confirm if QSRR Automator worked on the complicated data often found within an untargeted metabolomics analysis, we simplified the development by using a standard mix of 400 compounds. We used this standard mix on five different chemistries of HILIC column to test predictability of the results of each column chemistry. After filtering as described in the Methods Section, each column resulted in 230–260 identified compounds available for testing which are listed in Table S6. Samples were split into a training and test split and models were created. Five training/test splits were used each with 3 replicates to ensure that this analysis was truly representative of the performance of QSRR Automator. The prediction performance on the testing set is shown in Figure 4 with more data on the individual models present in Table S7.

As can be seen in Figure 4, 68–84% of predictions are within one minute of their true value, depending on column type, and 85–96% of all points are within two minutes, again depending on column type. With chromatography runs of this length this should be sufficient to add confidence to identifications based on exact mass. As with the lipids, error is evenly distributed around zero in the histograms and bias in individual training/test splits is limited to the individual split. Therefore, bias is based on training set used, not a software bias.

Further differences between column types can be observed based on the models. The most obvious comparison between models, aside from predictive ability on the test set, is the molecular features that are used for prediction. If a feature is used consistently in all models for a given column, it is likely critical to predicting retention times on that column. Conversely, if a molecular feature is used in only one or two of the fifteen models for a given column, it is likely a quirk of the random number seeds used in model generation or the differences between the training sets used. This comparison is performed in Table S8. Within all models generated for a given column, there is a subset of 10–20 molecular features that are present in most or all of the models, with many more features being present in a smaller number of models. An example of one of these critical features is SLogP, which is present for every model for every column. SLogP represents the octanol partition co-efficient. This is a measure of hydrophobicity, which is to be expected in polar columns such as HILIC. While the rest of the critical 10–20 features vary from column to column, most of these features are related to polarity or which functional groups are present, which is again expected and should be slightly different based on column chemistry and different training sets. While the number of features is consistently large, it seems this is mostly a quirk of random chance, with a core of critical and relevant features still being used. Finally, in Table S7, the SVR model is most often preferred though occasionally Random Forest models are chosen. This shows the complexity of predicting HILIC since the simpler LR model was never chosen.

Overfitting is a concern in fitting machine learning models. Using too many features in the model can result in excellent fitting of the training set, at the cost of worse predictions on any new data. Since we are using established algorithms and cross-validations, feature selection should be robust to problems. However, it is still a concern especially with the number and variation of features observed. HILIC chromatography was used to test this due to its poorer prediction performance in general so differences in performance are easier to observe. To analyze this possibility, we created models using QSRR Automator on the same test/training splits as the unrestricted model selection, and forcing the final model to use only a particular number of features. The results on the appropriate test sets were compared, with a summary of results in Table S9. A comparison of the results shows that, on average, the unrestricted selection of features has less average prediction error than restricting the number of features. While this will reduce conclusions drawn about chemical interactions with the column from the most predictive features, the resulting models will accurately predict retention time. Further we have examined the features used in Table S8. While there are many features used when considering all 15 models created for each column condition, however very few features are used in each model. There are only 4–15 features used in all models for their column. While there are more features that occur in all but one or two replicates, the vast majority of features are used in only one or two replicates. This indicates there are a core group of features consistently chosen that are truly necessary, while the rest are quirks of feature selection. In combination with the lack of prediction improvement on unseen data when limiting numbers of features it is unlikely to harm the user’s predictions to a large degree unless using vastly different compounds from the training set.

**Figure 5:**
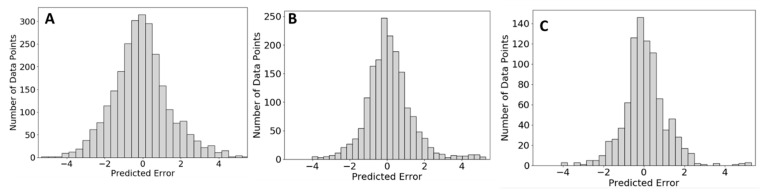
Analysis of error with smaller training sets. This figure shows the data from HILIC-Z with different training set sizes. (**A**). 60 compounds in training sets (**B**). 120 compounds in training set (**C**). 180 compounds in training set. The 180 compound (**C**) uses the same data as Figure 4C.

Not all labs have access to a mix of 180–200 standards they can confidently identify to use on machine learning calibration. To show how QSRR Automator performs on more limited training sets. Figure 5 shows the results of predicting compounds with training sets of size 60 compounds, 120 compounds, and 180 compounds from the HILIC-Z column as an example. Splits, replicates and randomization were performed as for Figure 4. There are more predictions in the smaller training sets due to the compounds not used for the training sets being moved to the test sets. As can be seen, there is more spread to the error when fewer compounds in the training sets, but the predictions are still clustered around 0 min and the rough shape of the histogram is similar.

#### 2.2.3. Comparison of QSRR Automator Models on HILIC Columns

With the different results shown in Figure 4, it is reasonable to attempt to determine which HILIC column is most easily modeled by QSRR Automator. An examination of Figure 4B quickly reveals that the BEH-Amide column as the lowest spread in absolute prediction error. However, BEH Amide is the column with the shortest gradient, which allows less time for errors. An analysis of error alone also does not account for things such as the average number of features the models use, with fewer features generally being a protection against overfitting. Relevant error metrics are listed in Table 3.

As can be seen in Table 3, the prediction error centers around 0. BEH Amide have the best accuracy, while the HILIC-z column generally produces models with the fewest features, and so should be the most robust to overfitting.

## 3. Discussion

It is important to acknowledge the limitations of QSRR Automator observed while collecting these results. Like any statistical model, extrapolating beyond the retention times observed in the training set may lead to inaccurate results. Furthermore, though the training set contained a wide variety of chemically diverse compounds, compounds that are different from the training set in structure will likely not be predicted accurately. Finally, the more complex the compounds the larger the training set must be to accommodate the differences. Lipids give better predictions than metabolites with a similar sized training set because lipids typically share a common backbone, similar head groups and fatty acid building blocks, as opposed to the wide variety of sizes and functional groups found among metabolites.

Even with these limitations, the prediction does place the majority of predictions within one minute of their true value (approximately 6% of the run time), with almost all predictions within 2 min (approximately 11–22% depending on the column). In all tests QSRR Automator performs comparably to published methods. While inferior to a dedicated bioinformatician, it will create and store many models in a fast and user-friendly manner. Similarly, tests on in-house data performed well. Predictions are within one or two minutes depending on training set and column. While insufficient to separate compounds that very nearly co-elute, such as leucine and isoleucine, it is sufficient to improve confidence in exact mass identifications and differentiate between clearly separated compounds of the same mass. QSRR Automator can aid investigators with the retention time prediction of multiple columns and conditions.

It is difficult to fully compare different chromatography columns to each other due to the widely varying compounds used or observed and the different HPLC set-ups required. However, a few conclusions can be reached. In both the published and in-house results lipidomics prediction is superior to metabolomics in the amount of error present in the predictions on the test set. When comparing in-house metabolomics data, we observed that the BEH Amide column had the least error in prediction but required the most features for its models, while the HILIC-z column generally required the fewest features, so is likely most robust to overfitting. Which column is better for a given application will depend on the compounds being considered. For example, it may be desirable to choose a column where target compounds are known or predicted to have widely spaced elution times, regardless of the prediction error the models for such a column generally contain.

Future directions for this work include attempting to limit the number of features generated by the models. Moreover, testing compounds run at the beginning and end of a column’s life to determine how a model generated at the start of a column’s life predicts compound behavior at the end of the column’s life.

## 4. Materials and Methods

### 4.1. QSRR Automator Software

#### 4.1.1. Software Used in the Creation of QSRR Automator

QSRR Automator was created using the Python programming language. Molecular descriptors were determined by using the Mordred software package [13] which uses the rdkit package [14]. Machine learning operations were performed using the sci-kit learn package [15].

#### 4.1.2. QSRR Automator Workflow

**Figure 6:**
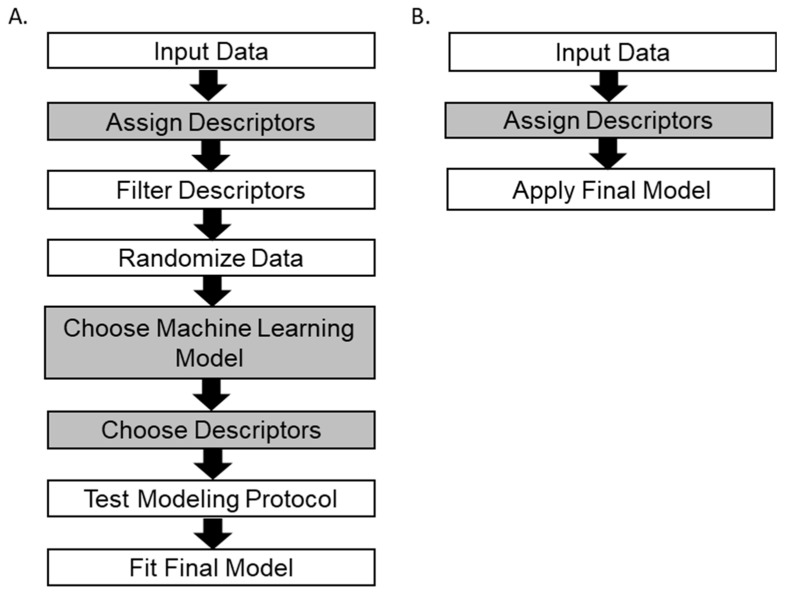
QSRR Automator workflow. Gray cells are steps where the user has the option to force a specific choice, such as by providing descriptors or adjusting settings. (**A**). Generating a model. Data are requested from the user, descriptors are assigned and filtered. Data are randomized. A machine learning model is chosen and tested with a cross-validation. All data are used for the final model. (**B**) Using a previously generated model as in Panel (**A**) retention time for new data can be predicted. Data are provided, descriptors provided if necessary and retention times are predicted by the model in use.

The general workflow is given in Figure 6. The user provides training data which consist of a name for each compound, the structure in the form of a Simplified Molecular Input Line Entry System (SMILES) text string [16] and a retention time. A template can be generated by QSRR Automator to make the input file easier to create. The user can provide chemical descriptors or they will be calculated from the SMILES using the Mordred software package [13]. These descriptors are broadly structural or electrical in nature. Structural features include amounts of various functional groups, amount and size of ring systems, elemental composition, and the number of sp3 hyridized carbons vs. the number of sp hybridized carbons. Electrical features involve special orbital effects such as aromaticity, and multiple calculations of electronegativity. All descriptors are rather basic calculations, unlike more complex fingerprint combination of features that other calculators sometimes employ. Mordred will calculate approximately 1600 features. Following the example of other QSRR models for lipidomics and metabolomics [2,6] the descriptors are filtered. Descriptors with too many duplicate values (default 90% of compounds have the same value), too many missing values (default 75% of samples missing a descriptor) or high correlation to related descriptors (default r = 0.9 or more) are removed. After these filters, approximately 400 features remained for metabolite data sets, and approximately 300 features remained for the lipid data set.

After collecting all valid descriptors, the data order is randomized. The machine learning methods of Linear Regression (LR), Random Forest, and Support Vector Regression (SVR) which have been used in previously published QSRR models [1,2,6,7] are available in QSRR Automator. If the user did not specify a method, all will be attempted and the best selected. The chosen model may be different for the same information run multiple times due to random data splitting, but all resulting models are of similar in predictive ability.

A data scaling step, a feature selection step if relevant, and the machine learning step were placed into a scikit-learn pipeline. This pipeline is then fed into a cross-validation (5-fold for all analyses in this paper). The pipeline will be performed on the training set of the cross-validation and then the resulting model will be used on the on the cross-validation fold’s test set. Performance is evaluated on absolute time error and the r^2^ of the resulting model on that fold of the cross-validation’s test set. Feature selection is a performed by a recursive feature elimination cross validation using a random forest regressor. If the user has specified that QSRR Automator should choose the model or if the user chosen model is SVR, a grid search cross validation is performed. If SVR is considered, the hyperparameters being tuned are C and gamma. If the model is being chosen as well, hyperparameters include which model to use (LR, RF, or SVR) as well as SVR’s gamma and C values. The average and median r^2^ of this step along with the mean absolute error will be reported to the user so they can evaluate if this method will likely work well on their data.

After various cross-validations are completed all of the data will be used to create a final model. This will still use the same scikit-learn pipeline as the initial step, without the external cross-validation (the cross-validations for feature selection and model selection are still present if the settings require them). How accurately the final model will predict new retention times can be roughly estimated by the values from the cross-validations so the final model can use all of the initial data. The mean and median or r^2^ are provided for the cross validations, as well as the mean absolute time error of the prediction and its standard deviation for the cross-validation. The final graph provided shows the model made using all the data (with its final r^2^ and absolute error) is also presented (Figure 1B). If the user accepts the model, it will be saved for later use. The user may also adjust settings and attempt to build the model with different random numbers if desired.

For predicting unknown retention times, the user must provide a template file with compound names and SMILES. If no descriptors are present in the template file, QSRR Automator will again attempt to create them. The currently loaded model will be used to predict the retention times based on the descriptors and will write them to an output file.

### 4.2. Comparison to Previously Published Data

Data from previously published lipidomics or metabolomics QSRR models were collected [1,2,6,7]. Basic details on these data-sets are provided in Table 1.

By default, QSRR Automator was set to run a 5-fold cross-validation and a 5-fold internal grid search cross validation. Allowed to compare random forest (RF), linear regression (LR), and Support Vector Machines for Regression (SVR). RF had 500 trees. SVR used the Radial Basis Function (rbf) kernel and was allowed to use C and gamma values between 0.001 and 1000. Feature selection was done using RF with 500 trees.

QSRR Automator was compared to the test set used for final validation of the published method for the data sets where such were supplied [1,2,7]. A direct comparison worked in two cases. In the paper by Creek et al. [2] data was heavily filtered after prediction. In many cases QSRR Automator chose a different one of a number of duplicates or gave a “good” prediction according to the filters for a metabolite discarded by the published model. To ensure the comparisons were fair, QSRR Automator was used on all potential peaks (any missing or uninterpretable SMILES were found using the pubchem database [17]) and the results were filtered according to the criteria of Creek et al. [2].

### 4.3. Tests on in-House Data

#### 4.3.1. Lipid Extraction

Red blood cells were extracted using a modified Matyash procedure [18]. All solutions used were pre-chilled on ice prior to extraction. Red blood cell aliquots (50 µL) were transferred to 13 × 100 glass vials, then extracted using a solution of 225 µL MeOH which contained internal standards (Avanti SPLASH LipidoMix at 10 µL per sample) and 750 µL MTBE (methyl tert-butyl ether). The samples were sonicated for 2 min followed by a rest on ice for 1 h with occasional vortexing. An addition of 188 µL dd-H_2_O was made to induce phase separation. After centrifugation at 3000 *g* for 5 min at 4 °C, the upper phases are collected and evaporated to dryness under a gentle nitrogen stream at room temperature. Lipid samples were reconstituted in 250 µL IPA (isopropyl alcohol) and transferred to an LC-MS vial with insert for analysis. Concurrently, a process blank sample and pooled quality control (QC) sample is prepared by taking equal volumes (~50 µL) from each sample after final resuspension.

Lipid extracts were separated on a Waters Acquity UPLC CSH C18 1.7 µm 2.1 × 100 mm column maintained at 65 °C connected to an Agilent HiP 1290 Sampler, Agilent 1290 Infinity pump, equipped with an Agilent 1290 Flex Cube and Agilent 6530 Accurate Mass Q-ToF dual AJS-ESI mass spectrometer. For positive mode analysis, the source gas temperature was set to 225 °C, with a drying gas flow of 11 L/min, nebulizer pressure of 40 psig, sheath gas temp of 350 °C and sheath gas flow of 11 L/min. VCap voltage was set at 3500 V, nozzle voltage 1000 V, fragmentor at 110 V, skimmer at 85 V and octopole RF peak at 750 V. For negative mode analysis, the source gas temperature was set to 300 °C, with a drying gas flow of 11 L/min, a nebulizer pressure of 30 psig, sheath gas temp of 350 °C and sheath gas flow 11 L/min. VCap voltage was set at 3500 V, nozzle voltage 2000 V, fragmentor at 100 V, skimmer at 65 V and octopole RF peak at 750 V. Samples were run in a randomized order in both positive and negative ionization modes in separate experiments acquiring with the scan range *m*/*z* 100–1700. Mobile phase A consisted of ACN:H_2_O (60:40 *v*/*v*) in 10 mM ammonium formate and 0.1% formic acid, and mobile phase B consists of IPA:ACN:H_2_O (90:9:1 *v*/*v*) in 10 mM ammonium formate and 0.1% formic acid. The chromatography gradient for both ionization modes started at 15% mobile phase B then increased to 30% B over 2.4 min, then increased to 48% B from 2.4–3.0 min, then increased to 82% B from 3–13.2 min, then increased to 99% B from 13.2–13.8 min where it was held until 16.7 min and then returned to the initial condition and equilibrated for 5 min. Flow was 0.4 mL/min throughout, injection volume was 3 µL for positive and 10 µL negative mode. Tandem mass spectrometry is conducted using the same LC gradient at collision energy of 25 V.

Results from LC-MS experiments were collected using Agilent Mass Hunter (MH) Workstation and analyzed using the software packages MH Qual, MH Quant, and Lipid Annotator (Agilent Technologies, Inc., Santa Clara, CA, USA). Results from the positive and negative ionization modes from Lipid Annotator were merged then split based on the class of lipid identified. Lipid targets are normalized based on the ratio to the internal standards and parsed based on the following criteria: lipids with relative standard deviations (RSD) less than 30% in QC samples and with background AUC counts in process blanks less than 30% of QC are used for data analysis.

SMILES were collected from PubChem [17] and LipidMaps [19]. Due to the potential ambiguity of lipid identification (locations of double bonds and occasionally tail lengths) the most biologically relevant lipids were selected. If this caused error in modeling or prediction they were minimal based on the results. Lipids were excluded if they were the only member of a lipid class that was vastly different from the other lipid classes or if the double bond locations had no consensus in PubChem or LipidMaps and would thus be required to be placed at random.

Training and test sets were made using the following method. All data was randomized using Microsoft Excel (2016). The first 75% of the randomized data was designated as the training set and the remaining 25% was designated as the test set. Each training set was used to generate 3 models using QSRR Automator, using 5-fold cross-validation and a 5-fold internal grid search cross validation. QSRR Automator selected the machine learning algorithm used to create its prediction model from the following: random forest (RF), linear regression (LR), and Support Vector Machines for Regression (SVR). Whichever algorithm performed best in the grid search cross validation was used for that model. RF used 500 decision trees for both feature selection and the final algorithm. SVR used the rbf kernel and was allowed to use C and gamma values between 0.001 and 1000. Test set values were predicted for each of the 3 models generated. Overall, 5 test/training set splits were created for the data from each column.

When comparing predicted retention times to observed retention times, any compounds in the test set with an observed retention time later than the latest observed retention time in the training set or before the earliest observed retention time in the training set were discarded. Extrapolating beyond the bounds of the training set can easily lead to inaccurate predictions. While extrapolation did not cause inaccurate predictions in the lipidomics data, they were few extrapolated compounds and removing them allowed consistency with the metabolomics section below for which extrapolation caused large prediction inaccuracies. There were two compounds for which this occurred, one in split 3, and one in split 4.

#### 4.3.2. Metabolomics Data

A sample consisting of over 400 standards was analyzed by mass spectrometry. An Agilent 6545 UPLC-Q-ToF (Agilent Technologies, Inc.) run in both positive and negative modes was used for analysis. Separation was achieved using a Sequant ZIC-pHILIC, ZIC-cHILIC (Millipore Sigma, Burlington, MA, USA), iHILIC-fusion (HILICON, Umeå, Sweden), HILIC-z (Agilent Technologies, Inc.), and BEH amide (Waters Corportation, Milford, MA, USA). A Krudkatcher, for HPLC columns, or Krudkatcher Ultra (Phenomenex, Torrence, CA, USA), for UPLC columns, was used as a pre-column. The column compartment was heated 40 °C. An amount of 1 µL of each standard mix was injected per run. For the ZIC-pHILIC, ZIC-cHILIC, iHILIC-fusion, and HILIC-z An initial concentration of 95% ACN with 5% ddH_2_O (buffer B) and 1% 50 mM ammonium carbonate, for ZIC-pHILIC and HILIC-z, or 50 mM ammonium formate, for all other columns, in ddH_2_O (buffer A) was held for 1 min at a flow rate of 0.15 mL/min. B was decreased to 20% over 17 min and held for 2 min. B was returned to starting conditions over 0.1 min, and the system was allowed to re-equilibrate for 10 min between runs. For the BEH amide an initial concentration of 95% ACN with 5% ddH_2_O (buffer B) and 1% 50 mM ammonium carbonate in ddH_2_O (buffer A) was held for 1 min at a flow rate of 0.3 mL/min. B was decreased to 20% over 9 min and held for 2 min. B was returned to starting conditions over 0.1 min, and the system was allowed to re-equilibrate for 10 min between runs. For MS analysis, the source gas temperature was set to 250 °C, with a drying gas flow of 12 L/min, nebulizer pressure of 35 psig, sheath gas temp of 325 °C and sheath gas flow of 11 L/min. VCap voltage was set at 3500 V, nozzle voltage 0 V, fragmentor at 100 V, skimmer at 65 V and octopole RF peak at 750 V.

Compounds were identified by comparison of MS/MS fragmentation results to the METLIN database, the Human Metabolome Database (HMDB), or predicted lipid fragmentation from LipidMaps [19,20,21]. For compounds without MS/MS fragmentation, the *m*/*z* value was required to correspond to a unique peak in the correct pool. If such a peak was not observed or there were multiple peaks for that *m*/*z* value in the appropriate pool, the compound was dropped from consideration. Do note that this resulted in different compounds being observed in different columns. Observed retention time standard deviation (for compounds observed in multiple replicates) was 0.005–0.2 for most compounds. Compounds with phosphates spread across a wide time area in HILIC columns and so could have standard deviations ranging from 0.4–1 min.

Compounds were further trimmed by several metrics. If multiple compounds were chiral versions of each other, all but one was removed. Moreover, compounds with a single defining feature not well represented in other compounds, such as E, Z double bond isomers and long chain fatty acids, were removed due to poor ability of any machine learning model to predict features poorly represented in the training set. Compounds used for further analysis and their retention times are presented in Table S3. This resulted in about 240–260 compounds per column.

Training and test sets were made using the following method. All data was randomized using Microsoft Excel (2016). The first 75% (180–200 compounds) of the randomized data was designated as the training set and the remaining 25% (60–70 compounds) was designated as the test set. Each training set was used to generate 3 models using QSRR Automator, using 5-fold cross-validation and a 5-fold internal grid search cross validation. QSRR Automator selected the machine learning algorithm used to create its prediction model from the following: random forest (RF), linear regression (LR), and Support Vector Machines for Regression (SVR). Whichever algorithm performed best in the grid search cross validation was used for that model. RF used 500 decision trees for both feature selection and the final algorithm. SVR used the rbf kernel and was allowed to use C and gamma values between 0.001 and 1000. Feature selection was done using RF with 500 trees. Test set values were predicted for each of the 3 models generated. In total, 5 test/training set splits were created for the data from each column.

When comparing predicted retention times to observed retention times, any compounds in the test set with an observed retention time later than the latest observed retention time in the training set or before the earliest observed retention time in the training set were discarded. Extrapolating beyond the bounds of the training set can easily lead to inaccurate predictions. For metabolomics, extrapolated compounds were consistently problematic regardless of how well the same compound could be predicted using training sets with a wider retention time range. Any large error in prediction as determined by large absolute error or high Cook’s D value were re-examined. If problems were found, such as user error, a large peak masking the correct peak, or expert knowledge confirming the peak was observed at a retention time far removed from where it should be, the peak was corrected or removed as appropriate. The analysis was re-done from creating the test/training splits. All other methods were identical. For compounds removed for extrapolation or other errors, 4 compounds were removed from BEH-Amide, 4 compounds were removed from CHILIC, 5 compounds were removed from HILIC-Z, 5 compounds were removed from iHILIC, 3 compounds were removed from PHILIC.

For Figure 5, HILIC-Z 5 splits were created with 60 or 120 features and the rest of the compounds were predicted as with methods described above.

#### 4.3.3. Limited Feature Analysis

QSRR Automator created models with 6, 9 and 12 features on the test/training splits and settings form the metabolomics analysis. Results of predictions on the appropriate test sets on these models was compared to the predictions from the models created using larger numbers of features.

## Figures and Tables

**Table 1:** Comparison to published data-sets. This table provides details on the various data-sets used and how the QSRR Automator models compare. Data-Set provides an abbreviated name, -omics type is whether the compounds are from a lipidomics or metabolomics study, Column is whether a reverse phase (RP) or Hydrophilic Interaction Chromatography (HILIC). Published Model and QSRR Automator Model is the machine learning model used in each analysis, and Published # of Features and QSRR Automator # of Features are how many molecular descriptors were used in the appropriate final model.

Data-Set	-omics Type	Column	Published Model	Published # of Features	QSRR Automator Model	QSSR Automator # of Features
RP_Lipid [1]	Lipidomics	RP	SVR	12	SVR	11–31
RP_Met [7]	Metabolomics	RP	MLR	3	RF or SVR	11–246
HILIC_MLR1 [7]	Metabolomics	HILIC	MLR	3	SVR	21–146
HILIC_MLR2 [2]	Metabolomics	HILIC	MLR	6	SVR	14–44
HILIC_RF [6]	Metabolomics	HILIC	RF	4	RF or SVR	14–294

**Table 2:** Best fit least squares lines to the data in Figure 2. In an ideal fit y = x and r^2^ = 1. Rp_lipid does not have an equation for one value that is provided in the paper (though they did many tests for various reasons). Data-set names are from Table 1.

Data-Set	Published Best Fit Equation	Published r^2^	QSRR Automater Best Fit Line	QSRR Automater r^2^
RP_Lipid	*n*/a	0.989	y = 0.9778x + 0.1736	0.9942
RP_Met	y = 0.8929x + 1.4018	0.7685	y = 0.8466x + 1.824	0.7935
HILIC_MLR1	y = 0.5825x + 1.1667	0.65	y = 0.5789x + 0.8424	0.5911
HILIC_MLR2	y = 0.8812x + 0.8575	0.8375	y = 0.7909x + 2.0814	0.7385
HILIC_RF	y = 0.9523x + 0.3217	0.8596	y = 0.7729x + 2.8072	0.6667

**Table 3:** Error metrics for QSRR Automator models from different HILIC columns. Elution time range is given as retention time of first observed compound–retention time of final observed compound.

	BEH Amide	cHILIC	HILIC-z	iHILIC	pHILIC
Median Prediction Error	0.009	0.044	−0.022	0.029	−0.025
Median Prediction % Error	0.18%	0.49%	0.59%	−0.28%	−0.29%
Average # of features per model	72.4	58	19	59.73	58.73
Elution time range (min)	1.1–8.8	2.0–15	1.8–12	1.6–13	1.5–18

## Supplementary Materials

The following are available online at https://www.mdpi.com/2218-1989/10/6/237/s1, Table S1: Filtering comparison of HILIC_MLR2 data used in Figure 2, Table S2: *p*-values of published predictions and QSRR Automator predictions compared to observed data, Table S3: Observed in-house lipids retention times, Table S4: Details of QSRR Automator models created for in-house lipid data prediction, Table S5: How often molecular features were used in in-house lipid prediction models, Table S6: Observed in-house metabolite retention times, Table S7: Details of QSRR Automator models created for in-house metabolite data prediction, Table S8: How often molecular features were used in in-house metabolite prediction models, Table S9: Comparison of predictive ability in limited feature selection vs. unrestricted feature selection on in-house metabolite data.

## Program Availability

For ease of use a exe file contained in a zip folder with necessary dependencies and an instruction manual are provided at https://github.com/UofUMetabolomicsCore/QSRR_Automator/releases/tag/v1_exe. If the source code is desired for examination or modification it can be accessed at https://github.com/UofUMetabolomicsCore/QSRR_Automator. Note that code has only been tested on Windows machines.
