# Supplementary material for: QSRR Automator: A Tool for Automating Retention Time Prediction in Lipidomics and Metabolomics
Source: Metabolites. 2020 Jun 9;10(6):237. doi: 10.3390/metabo10060237 (PMC7345697; doi:10.3390/metabo10060237)
Supplement: Supplementary file 1 [file metabolites-10-00237-s001.zip › Supplementary Figure Key.pdf]

Supplementary Figure Key

Table S1

Due to the unique situation of hilic_mlr2 requiring filtering QSRR Automator data and the large number of the points making interpretation of Figure 4 D and E difficult, this table was created. "Peaks to predict" is the number of peaks observed in the mass spectrometry run with potential metabolites assigned. "Compounds after filtering" is the number of compounds observed after all filtering. "Training standards observed" is the number of standards used in the model training set that were potentially observed in the data set and which were definitely in the standards data set. These should be predicted well since the model was trained with them. Training Standards after filtering is the number of these standards that remained after filtering.

Table S2

Statistical tests to determine if there is a statistical difference between QSRR Automator and the published data or between either model and the target. Most exhibit no statistical difference. Hilic_mlr2 is statistically significant, but this may be a result of the large number of samples present which tends to decrease the p-value. All t-tests are paired t-tests except those comparing hilic_mlr2 to QSRR Automator directly. Since the compounds measured were not the same, a paired t-test is not appropriate so a standard t-test was used instead.

Table S3

Table of all compounds observed in the lipidomics dataset. Compound is the compound abbreviation and the C18 Time (minutes) column is the retention time the m/z was observed at.

Table S4

Table contains the best fit lines on the test sets from all models used in the Lipidomics dataset. Contains information on which split and replicate number, the number of features used in model creation and the machine learning model used (SVR for Support Vector Machines). If an SVR model was used the C and gamma variables are specified and the slope, intercept and r^2 of the best fit of an observed retention time vs. predicted retention time graph for the test set data is provided. At the end of each column section is an average of the # of features used and the best fit line metrics for all data from that column.

Table S5

Table contains the number of features used in all models used in the Lipidomics data set. The feature names come from the Mordred software package and are detailed on http://mordred-descriptor.github.io/documentation/master/descriptors.html. The numbers next to the feature names correspond to the number of models out of 15 (5 test/training splits with 3 replicates each) the feature was used in.

Table S6

Table of all compounds from standard mix observed in at least one column condition in the in-house metabolomics data-set. The retention time of the highest point of each compound's peak is recorded. Peaks are confirmed by ms/ms or exact mass. Not all compounds are observed in all columns, and in some columns identification is not certain and so was not reported.

Table S7

Table contains the best fit lines on the test sets from all models used in the Metabolomics dataset. Contains information on the column, which split and replicate number, the number of features used in model creation and the machine learning model used (SVR for Support Vector Machines, RF for Random Forest). If an SVR model was used the C and gamma variables are specified and the slope, intercept and r^2 of the best fit of an observed retention time vs. predicted retention time graph for the test set data is provided. At the end of each column section is an average of the # of features used and the best fit line metrics for all data from that column.

Table S8

Table contains the number of features used in all models used in the Metabolomics data set. Top row is the column used. The feature names come from the Mordred software package and are detailed on http://mordred-descriptor.github.io/documentation/master/descriptors.html. The numbers next to the feature names correspond to the number of models out of 15 (5 test/training splits with 3 replicates each) the feature was used in.

Table S9

Comparison of Limited Feature Selection vs. Unrestricted Feature Selection for each column. Test/Training split data sets were the same in both cases. Slope, intercept and r^2 are from a graph comparing observed retention time vs. model prediction time for the test set. The error values are averages or medians of all predicted values – observed values in a split. Column type, splits, and feature selection type are indicated.
